# Piloting an infrastructure for the secondary use of health data: learnings from the HealthData@EU Pilot

**DOI:** 10.1093/eurpub/ckaf073

**Published:** 2025-09-10

**Authors:** Christian Fynbo Christiansen, Persephone Doupi, Nienke Schutte, Damir Ivanković

**Affiliations:** Department of Clinical Epidemiology, Aarhus University and Aarhus University Hospital, Aarhus N, Denmark; Department of Clinical Medicine, Aarhus University, Aarhus N, Denmark; CONNECT—Center for Clinical and Genomic Data, Aarhus University Hospital, Central Denmark Region, Denmark; Data and Analytics Department, Finnish Institute for Health and Welfare, Helsinki, Finland; Innovation in Health Information Systems Unit, Scientific Directorate Data Governance, Sciensano, Belgium; European Public Health Association (EUPHA), Utrecht, The Netherlands

## Introduction

The European Health Data Space (EHDS) regulation creates a health-specific ecosystem for both primary and secondary use of health data. HealthData@EU—the novel cross-border technical infrastructure for secondary use of electronic health data will be crucial for achieving the ambitious goals of the EHDS.

In 2022, the “HealthData@EU pilot project,” co-funded under the EU4Health-framework (GA nr 101079839), brought together 17 partners including potential Health Data Access Bodies’ (HDABs) candidates, health data sharing infrastructures, and European agencies in order to build and test a pilot version of the HealthData@EU infrastructure and provide recommendations for metadata standards, data quality, data security, and data transfer to support development of the EHDS cross-border infrastructure.

This editorial and the other manuscripts presented in this Special EJPH Supplement will provide readers with insights from real-life scenarios that follow the research user journey and highlight the challenges of health research, as well as the solutions the EHDS can provide.

## The impact of the HealthData@EU

Multinational collaboration on large health data is needed to support evidence-based policy making and planning toward addressing the health challenges Europe is currently facing. The Joint Action Towards the European Health Data Space (TEHDAS, 2020–23) showed that Member States vary widely in how they organize and manage their health data, as well as in their preparedness to facilitate secondary use of health data [1].

As the population is aging and the prognosis of chronic diseases is improving, more European citizens are living with one or more chronic diseases. In addition, emerging infections, such as the corona virus disease 2019 (COVID-19) pandemic and influenza outbreaks, underscore the need for cross-border collaboration on public health challenges. Finally, improving care for rare diseases requires large data sets that can only be achieved by multinational collaboration. HealthData@EU provides researchers with the infrastructure to find and access data from multiple sources, in order to provide the best scientific evidence that can underpin policy decisions [2].

## The data user benefits of HealthData@EU

Data users of HealthData@EU include a wide range of individuals and organizations such as researchers, private companies, regulators, and citizens. For the data users, the European Health Data Space aims to improve all aspects of the user journey—a simplified approximation of the steps envisioned for cross-border secondary use of data. After specifying the research question, the data user will proceed to the data discovery phase, to find the data needed to address the question. A central service will be the first point of contact; it will facilitate data discovery through metadata catalogs and redirect data requests to national nodes in the Member States. When appropriate data are identified, a data application can be sent through the central service of the HealthData@EU infrastructure to the potential national coordinating HDABs of Member States. Potential HDAB candidates can secure the privacy of individuals by giving access only to pseudonymized data through a secure processing environment. The results of the data analyses, i.e. aggregated data, can afterwards be extracted from the secure processing environment and shared for the specific purpose of use, e.g. in scientific publications.

## Overall learnings from the HealthData@EU Pilot project

Five use cases in the HealthData@EU Pilot project have assessed the feasibility of addressing important health questions across countries and the need for an infrastructure such as HealthData@EU. They also all demonstrated that clinical and organization-level knowledge about the data content, context, and quality is crucial to analyze and interpret data. Furthermore, the use cases also showed that access procedures and requirements for researchers to be able to process health data are not always clear and transparent, particularly in the context of genomic data [3]. By providing on a European level one entry point and one form for data access requests with clear turnaround times, the EHDS can aid in streamlining the user journey. Finally, as the EHDS regulation assigns many operative tasks to the potential future HDABs, for HealthData@EU Pilot project also provides recommendations for these bodies, including economic scenarios [4].

## Future outlook

With the European Health Data Space regulation that came into force 26 March 2025, it is time to disseminate the results and learnings from this key project to all EHDS stakeholders. In order for the EHDS to be fully operational and implemented, legislative and technical harmonization will be needed. Thus, establishing the HealthData@EU infrastructure will require substantial efforts in most countries and a tailored road map for the implementation. The efforts will, however, be rewarded by an added value to the nationally demanded evidence in support of health decision-making and innovation. Synergy between national needs and EU-level targets will be instrumental in facilitating the process.

Conflict of interest: None declared.
